# Protective effects of *Althaea officinalis* L. extract against *N*‐diethylnitrosamine‐induced hepatocellular carcinoma in male Wistar rats through antioxidative, anti‐inflammatory, mitochondrial apoptosis and PI3K/Akt/mTOR signaling pathways

**DOI:** 10.1002/fsn3.3455

**Published:** 2023-05-26

**Authors:** Zhenqian Wang, Xiao Jiang, Long Zhang, Han Chen

**Affiliations:** ^1^ Department of General Surgery 905th Hospital of the Chinese People's Liberation Army Navy Shanghai P.R. China

**Keywords:** *Althaea officinalis* L., antioxidant, hepatocellular carcinoma, liver, *N*‐diethylnitrosamine

## Abstract

Hepatocellular carcinoma is the fourth cause of death due to cancer and includes 90% of liver tumors. Therefore, in this study, it was tried to show that *Althaea officinalis* L. flower extract (ALOF) can protect hepatocytes against *N*‐diethylnitrosamine (DEN)‐induced hepatocellular carcinoma. Totally, 70 Wistar rats were divided into seven groups (*n* = 10/group) of sham, DEN, treatment with silymarin (SIL; DEN + SIL), treatment with ALOF (DEN + 250 and 500 ALOF), and cotreatment with SIL and ALOF (DEN + SIL + 250 and 500 ALOF). At the end of the study, the serum levels of liver indices (albumin, total protein, bilirubin, C‐reactive protein, ALT, AST, and ALP), inflammatory cytokines (IL‐6, IL‐1β, IL‐10, and TNF‐α), and oxidants parameters (glutathione peroxidase [GPx], superoxide dismutase [SOD], catalase [CAT] activity along with nitric oxide [NO] levels) were evaluated. The level of Bax, Bcl‐2, Caspase‐3, p53, PI3K, mTOR, and AKT genes were measured. ALOF in cotreatment with SIL was able to regulate liver biochemical parameters, improve serum antioxidant indices, and decrease the level of proinflammatory cytokines significantly (*p* < .05). ALOF extract in both doses of 250 and 500 mg/kg in cotreatment with SIL caused a significant (*p* < .05) decrease in the p53‐positive cells and a significant (*p* < .05) increase in Bcl‐2‐positive cells. Therefore, ALOF was able to modulate the proliferation of cancer cells and protect normal cells through the regulation of Bax/Bcl‐2/p53 and PI3K/Akt/mTOR signaling pathways. It seems that ALOF can be used as a prodrug or complementary treatment in the protection of hepatocytes in induced damages caused by carcinogens.

## Graphical abstract

*Althaea officinalis* L. extract has remarkable antioxidant and anti‐inflammatory effects. *Althaea officinalis* L. extract can be used as a complementary treatment in the protection of hepatocytes in induced damages caused by carcinogens.

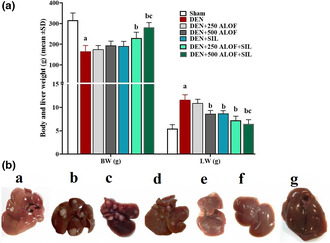

## 1 INTRODUCTION

Hepatocellular carcinoma (HCC) is the most common primary liver cancer with the incidence of 500,000–1000,000 new cases per year and 600,000 deaths per year directly related to it. The highest incidence rate of HCC is reported in Asia (55:100,000) and the lowest in Europe (5:100,000; Mak et al., 2020). Genetic changes including focal deletions at tumor suppressors (CDKN2B, p53, and PTEN) and focal amplifications at oncogenes (FGF19, MYC, and MET) along with changes in signaling pathways including PI3K/AKT, p53/p21, Wnt/β‐catenin, Notch, PI3K/AKT/mTOR, and JAK/STAT affect the metabolism, cell cycle, repair, angiogenesis, and cellular immunity of hepatocytes (Caruso et al., 2021). The main risk factors of HCC are viral hepatitis (hepatitis B and C), toxic factors (aflatoxins, chemotherapy drugs, alcohol, and nitrate‐based compounds), and metabolic (fatty liver disease, diabetes, and hereditary hemochromatosis) and immune‐related disorders. Some factors such as proinflammatory cytokines (IL‐6, IL‐1β, IL‐10, and TNF‐α), reactive oxygen species (ROS), and reactive nitrogen species (RNS) also cause the development of liver tumors by providing tumor microenvironment (Chen et al., 2020).

Nitrosamines or *N*‐nitrosamines are organic carcinogen compounds with the chemical formula R_2_N–N=O, which are produced during the reaction of nitrous acid (HNO_2_) and secondary amines. These compounds are produced in tobacco, smoke from car combustion, cosmetics, canned food products, and cooking frying products (meat, bread, and vegetables). Various types of nitrosamines including *N*‐nitrosonornicotine, 4‐(methylnitrosamino)‐1‐(3‐pyridyl)‐1‐butanone, *N*‐nitrosodimethylamine, *N*‐nitrosodiethylamine, 4‐(methylnitrosamino)‐1‐(3‐pyridyl)‐1‐butanol, *N*‐nitrosoanabasine, and *N*‐diethylnitrosamine (DEN) have been detected in food products (Park et al., 2015). Alkylating–methylating nitrosamines are compounds that cause damage to the DNA of normal cells. Studies have shown that rats exposed to tobacco‐derived nitrosamines developed various tumors in the colon, nose, lung, mouth, liver, esophagus, pancreas, and breast (Hoffmann et al., 2017). DEN is a carcinogen chemical substance used in the induction of various models of respiratory system, skin, gastrointestinal tract, and liver tumors. After the intraperitoneal injection or gavage of 10 mg/kg of body weight, the initial symptoms of liver toxicity (chronic inflammation and fibrosis) appear, and in doses lower than 30 mg/kg of body weight, the initial stages of induction, promotion, and progression of liver tumors are created (Memon et al., 2020). One of the reasons for tumor induction caused by DEN is the increased expression of G1/S‐phase regulatory proteins (such as cyclin‐dependent kinases) of hepatocytes. In addition, the subsequent biotransformation of DEN and alkylating production metabolites leads to DNA damage of hepatocytes. Following the cellular metabolism of DEN, ROS and RNS are produced. These agents reduce free radical scavenging/production ratio, and besides inhibiting endogenous antioxidant enzymes, they also attack cellular biological macromolecules (proteins, polyunsaturated fatty acids; Arboatti et al., 2018; Tolba et al., 2015).

DEN targets different apoptotic/autophagy/proliferative/differentiation pathways of liver parenchymal cells. PKB/AKT pathway is one of the pathways involved in the proliferation and differentiation of hepatocyte cells, and is inhibited by DEN (Hegazy et al., 2019). Meanwhile, DEN stimulates Wnt/β‐catenin and TGFβ1/Smads tumorigenic pathways of hepatocytes and accelerates their tumorigenesis process (Li et al., 2016). Another tumorigenic pathway of DEN is the stimulation of MEK1, 2/ERK1, 2/cyclin D1, and two signaling pathways along with the stimulation of PI3K/Akt/mTOR signaling pathways in hepatocytes. Studies have also shown that DEN inhibits the proliferation of normal and damaged hepatocytes through the stimulation of Bax/Bcl‐2/p53 mitochondrial apoptosis pathway (Li et al., 2022). DEN activates the ROS‐ and RNS‐induced p53‐dependent apoptotic pathway associated with cytochrome P450 of hepatocytes and triggers the apoptotic cascade (activating initiator caspases such as caspases 8 and 9 followed by activating the effector caspases such as caspases 3 and 6) by leaking cytochrome c from mitochondria (Mo'men et al., 2020). DEN and its metabolites stimulate Kupffer cells, neutrophils, and liver lymphocytes to increase the panel of proinflammatory cytokines (IL‐6, IL‐8, and IL‐1β) and inhibit the synthesis of anti‐inflammatory cytokines (IL‐10, IL‐4, IL‐2, and IL‐13; Chen et al., 2015). Therefore, the most suitable strategy for the treatment of HCC caused by DEN is the use of compounds that affect anti‐inflammatory, antioxidant, and proliferation/differentiation pathways. Different studies have shown that some plant extracts, including *Calendula officinalis* L., *Cassia fistula* L., *Salmo gairdneri* L., *Agaricus blazei* L., *Tinospora cordifolia* L., and *Punica granatum* L., due to their polyphenolic compounds (flavonoids and isoflavonoids), protect hepatocytes against DEN‐induced HCC through different pathways. The extracts of these plants contain different compounds such as daidzein, genistein, quercetin, kaempferol, catechin, resveratrol, curcumin, rutin, betulinic acid, and artemisinin, which suppress various tumors including HCC through anti‐inflammatory, antiangiogenic, and antiproliferative activities (Dhanasekaran et al., 2009; Dhingra et al., 2022; Kaur et al., 2019; Lee & Hong, 2011).

*Althaea officinalis* L. belongs to the Malvaceae family, which is found all over the world but is native to Eastern Europe, Northern Africa, and Asia. This plant grows in humid and semihumid areas and produces pinkish white flowers during July to September (Naveed et al., 2011). *Althaea officinalis* L. flower (ALOF) contain starch (25%–35%), mucilage (5%), saccharose (10%), pectins (11%), and the remaining include polyphenols (p‐coumaric acid, hypolaetin‐8‐glucoside, caffeic acid, isoquercitrin, kaempferol, genistein, daidzein, rutin, quercetin, and catechin), tannins, scopoletin, asparagine, coumarins, and phytosterols (Al‐Snafi, 2013; Sendker et al., 2017). Since ancient times, ALOF has been used as a seasoning for soups, teas, vegetables, and as an additive in food to treat dry coughs, flatulence, diabetes, hyperlipidemia, diarrhea, relief of high fever, and increase immunity against viruses and bacteria. In modern medicine, ALOF and its effective ingredients are used in the treatment of cardiovascular diseases, respiratory tract infections, and urolithiasis (Kianitalaei et al., 2019). In vitro studies have shown that ALOF extract with antiproliferative activity suppresses the proliferation of breast cancer cell line AMJ13, human lung cancer cell line A549, and prostate cancer cell line PC‐3 (Kadhum et al., 2021; Zhang et al., 2016). Studies have shown that the compounds present in this plant, such as kaempferol, genistein, p‐coumaric acid, daidzein, and quercetin act through PI3K/AKT/mTOR, MEK‐ERK, PI3K‐AKT/PKB, and AMPK/p53 signaling pathways, along with anti‐inflammatory and antioxidant effects. They suppress various breast, colon, lung, brain, and skin tumors in in vitro and in vivo models (He et al., 2011; Kaushik et al., 2019; Li et al., 2019). In the study of Morovatisharifabad et al. (2020), it was found that ALOF extract in doses of 300 and 600 mg/kg of body weight protected the function and structure of the liver against inflammatory/oxidative damage caused by diazinon toxin in hamsters (Morovatisharifabad et al., 2020). In this study, ALOF extract was able to improve the level of liver enzymes along with antioxidant parameters. For this purpose, in this study, we tried to evaluate the antitumor effects of ALOF against DEN‐induced hepatocellular carcinoma in Wistar rats through antioxidative, anti‐inflammatory, mitochondrial apoptosis and PI3K/Akt/mTOR signaling pathways.

## 2 MATERIALS AND METHODS

### 2.1 ALOF extract preparation

ALOF were dried at 25°C in a dark room after collection in early September and confirmed by the botanist. Dried ALOF (2000 g) was pulverized by a soil grinder (Humboldt, model number: H‐4199.5F). The resulting powder was then added to 2000 mL of 70% ethanol (30:70 distilled water [DW]:ethanol) and incubated for 72 h at 37°C in a dark room. The resulting mixture was passed through filter paper (Millipore, size number: 42) and condensed with an evaporator (Buchi, model number: R‐210). The final ALOF extract (570 g, efficiency rate of 28.5%) was dried at 37°C and stored at 4°C (Tiloke et al., 2019).

### 2.2 DEN and induction of hepatocarcinogenesis

The DEN (Sigma‐Aldrich) was purchased in dark bottles as a fresh solution (100 mg/L) and supplied to induce hepatocarcinogenesis following a previous method (Arboatti et al., 2018).

### 2.3 Animals and experimental design

Seventy male Wistar rats weighing 180 ± 20 g randomly were grouped into seven groups (*n* = 10/group) according to Table 1. The rats were kept for 72 h to adapt to the temperature, food, and water used in the study before starting the study. All rats were kept in propylene cages at a temperature of 25 ± 2°C, relative humidity of 50 ± 5%, and a 12/12‐h dark/light cycle. Standard rat pellets and tap drinking water were used for all groups. All procedures of maintenance and sacrifice of rats were carried in accordance with the national standards protocol and guidelines for the care of laboratory animals (NIH Publication 80‐23, 1996).

**TABLE 1:** Experimental design and animal grouping.

Group number	Group name	Treatment (dose and prescription manner)	Treatment period
1	Sham	500 μL PBS/i.p.	Days 7, 14, and 21
2	DEN	100 mg/kg DEN dissolved in 500 μL of PBS/i.p.	Days 7, 14, and 21
3	DEN + 250 ALOF	DEN + 250 mg/kg ALOF extract/p.o./p.d.	Days 7–42
4	DEN + 500 ALOF	DEN + 500 mg/kg ALOF extract/p.o./p.d.	Days 7–42
5	DEN + SIL	DEN + 0.5% SIL powder/p.o./p.d.	Days 7–42
6	DEN + 250 ALOF + SIL	DEN + 250 mg/kg ALOF extract + 0.5% SIL powder/p.o./p.d.	Days 7–42
7	DEN + 500 ALOF + SIL	DEN + 500 mg/kg ALOF extract + 0.5% SIL powder/p.o./p.d.	Days 7–42

Abbreviations: ALOF, *Althaea officinalis* L. flower; DEN, *N*‐diethylnitrosamine; i.p., intraperitoneal injection; p.d., per day; p.o., per oral; SIL, silymarin.

The total duration of the study was 42 days, and in groups 2–7, rats received 100 mg/kg DEN intraperitoneally (i.p.) at 8:00 a.m. on days 7, 14, and 21 of the study, and in groups 3 and 4, in addition to DEN, from days 7 to 42 the animals received 250 and 500 mg/kg of ALOF extract by gavage, respectively, every day at 15:00 p.m. In group 5, in addition to DEN, rats received 0.5% silymarin (SIL) powder by gavage from days 7 to 42 of the study every day at 15:00 p.m., and in groups 6 and 7, in addition to receiving DEN, rats received 250 and 500 mg/kg of ALOF, respectively, and SIL at 15:00 p.m. from days 7 to 42. To select the most appropriate dose of DEN and SIL, previous studies were used along with the pilot study. To choose the most effective and nontoxic dose of ALOF extract, LD_50_ (lethal dose) was also considered in addition to previous studies and a pilot study (Hage‐Sleiman et al., 2011; Imamoto et al., 2014; Talebi et al., 2014).

### 2.4 Acute toxicity test (LD_50_ ) of ALOF extract

To select a safe dose of ALOF extract, LD_50_ was used according to Lork's two‐step method. According to this method, first, 12 rats in three groups (*n* = 4/group) of doses of 50, 500, and 5000 mg/kg/i.p. received ALOF extract, and their general condition was monitored within 24 h. Doses in which any toxic symptoms or possible death were observed in rats were recorded. Then six rats in three other groups (*n* = 2/group) with doses of 10, 100, and 1000 mg/kg/i.p. ALOF extract was received, and the procedure was similar to the previous step. Finally, the lowest dose in which toxic symptoms or death were observed (*D*_L_), along with the highest dose (*D*_S_) in which rats did not have any toxic symptoms, were placed in the following formula and LD_50_ of ALOF extract was calculated (Feng et al., 2022).

$$LD_{50}=\left(D_{L}\times D_{S}\right)$$

In this study, during the evaluation of LD_50_, only in a dose of 5000 mg/kg ALOF extract caused nausea in rats, while no death occurred in this dose and lower doses. It seems that the nausea in the dose of 5000 mg/kg ALOF is caused by the irritation of the digestive system and this plant has no other toxic side effects.

### 2.5 ALOF extract total phenolic content (TPC)

Folin–Ciocalteu method was used to calculate ALOF extract TPC based on μg/mL gallic acid (GAE). In this method, 1 mL of Folin–Ciocalteu solution, 50 μL of ALOF hydroalcoholic extract, and 0.8 mL of 7.5% carbonate were mixed and incubated for 2 h at 37°C. Then, 50 μL of serial dilution of gallic acid (50, 75, 100, 200, and 400 μg/mL) was added to the mixture and incubated again for 2 h at 37°C. Finally, the absorbance of the resulting mixture was read at a wavelength of 765 nm by spectrophotometer (Shimadzu Co., model number: UVmini 1240; Hmamou et al., 2022).

### 2.6 ALOF extract total flavonoid content (TFC)

Miliauskas method was used to calculate ALOF extract TFC based on mg/mL. In this method, 0.1 mL of 1 M potassium acetate, 50 μL of ALOF hydroalcoholic extract, 2.5 mL of aluminum trichloride reagent 20 mg/mL, and 2.8 mL of distilled water (DW) were mixed and incubated for 1 h at 37°C. Then, 2.5 mL of serial dilution of rutin (0.025, 0.037, 0.050, and 0.075 mg/mL) was added to the mixture and incubated again for 40 min at 37°C. Finally, the absorbance of the resulting mixture was read at a wavelength of 415 nm by spectrophotometer (Shimadzu Co., model number: UVmini 1240; Hmamou et al., 2022).

### 2.7 ALOF extract 2,2‐diphenyl‐1‐picrylhydrazyl (DPPH) radical scavenging activity

Brand and Williams method was used to calculate ALOF extract DPPH based on μmol Trolox equivalent. In this method, 100 μL serial dilution of ALOF hydroalcoholic extract (0.025, 0.050, 0.075, 0.100, 0.125, and 0.150 mg/mL) and 3.5 mL DPPH radical solution 60 μM were mixed and after incubation at 25°C for 60 min, the absorbance of the resulting mixture was read at a wavelength of 515 nm by a spectrophotometer (Shimadzu Co., model number: UVmini 1240; Hmamou et al., 2022).

### 2.8 Ferric reducing antioxidant power (FRAP) assay

Benzie and Strain method was used to calculate ALOF extract total antioxidant capacity (TAC) based on FRAP method (mmol) Fe^2+^‐2,4,6‐tripyridyl‐s‐triazine (TPTZ). In this calorimetric method, which is based on the conversion of Fe^3+^‐TPTZ (yellow color) to Fe^2+^‐TPTZ (blue color), first 3 μL of FRAP solution, 1 mL of 40 mM TPTZ, 50 μL of ALOF hydroalcoholic extract, and 180 μL of DW were gently mixed and then incubated for 15 min at 37°C. The absorbance of the resulting mixture after centrifugation (15,000*g* for 10 min) was read at a wavelength of 593 nm by a spectrophotometer (Shimadzu Co., model number: UVmini 1240; Hmamou et al., 2022).

### 2.9 Analysis of serum‐related parameters

#### 2.9.1 Liver biochemical parameters

To evaluate the functional factors of the liver, on the 42nd day of the study, after performing the preanesthesia/anesthesia protocol with the help of 15 mg/kg ketamine 10% and 80 mg/kg xylazine 2%, an incision was made from the xiphoid area on the thorax and blood was taken from the heart. The serum was then separated and the level of aspartate aminotransferase (AST; catalog number: NBP2‐69877), alanine aminotransferase (ALT; catalog number: KA1294), alkaline phosphatase (ALP; catalog number: NBP2‐68196), albumin (ALB; catalog number: NBP2‐60483), total protein (TP; catalog number: DYC1663‐2), C‐reactive protein (CRP; catalog number: DY1744), and bilirubin (BIL; catalog number: NBP2‐69939) were measured with the help of Novus Biologicals commercial sandwich‐based ELISA (enzyme‐linked immunosorbent assay) kits (Novus Biologicals, LLC) and according to the manufacturer's recommendations (Liu et al., 2022).

#### 2.9.2 IL‐6, IL‐10, IL‐1β, and TNF‐α serum levels

In order to evaluate the anti‐inflammatory effects of ALOF extract, the levels of proinflammatory (IL‐1β [catalog number: RLB00], IL‐6 [catalog number: M6000B], and TNF‐α [catalog number: NBP2‐DY410]) and anti‐inflammatory (IL‐10 [catalog number: R1000]) cytokines were measured with the help of Novus Biologicals commercial sandwich‐based rodent‐specified ELISA kits (Novus Biologicals, LLC) and according to the manufacturer's recommendations. In short, after preparing standard dilutions, samples, and reagents, we first added 50 μL of assay diluent RD1‐21 to each well. Then, added 50 μL of standard, control, or sample per well and incubated for 2 h at room temperature. The wells are aspirated, and the wells are washed 5 times with 400 μL of washing buffer and we added 100 μL of rat IL‐6, IL‐10, IL‐1β, and TNF‐α conjugate to each well. After incubating the wells for 2 h at room temperature, added 100 μL of substrate solution to each well and incubated for 30 min at room temperature. Finally, after adding 100 μL of stop solution to each well and incubating the wells at room temperature for 30 min, the absorbance of the final mixture was read at 450 nm with an ELISA reader (Awareness Technology, Stat Fax ELISA reader, model number: 303 microwell readers; El‐Zayadi et al., 2020).

#### 2.9.3 Nitric oxide (NO) assay

The Griess colorimetric method was used to measure serum NO level as one of the most important serum factors indicating lipid peroxidation and oxidative stress. Briefly, 500 μL serum samples were added to 6 mg zinc oxide, and after mixing and centrifugation (10,000*g* for 15 min), the supernatant was transferred to 500 μL Griess solution. After incubation at 37°C for 60 min, the absorbance of the resulting mixture was read at a wavelength of 540 and 630 nm by a spectrophotometer (Shimadzu Co., model number: UVmini 1240; Akbari Bazm et al., 2020).

#### 2.9.4 Glutathione peroxidase (GPx), catalase (CAT), and superoxide dismutase (SOD) serum activity

A commercial Cusabio sandwich‐based rodent‐specified ELISA kit according to the manufacturer's recommendations was used to measure the serum activity of glutathione peroxidase (GPx; catalog number: CSB‐E12146r), catalase (CAT; catalog number: CSB‐E13439r), and superoxide dismutase (SOD; catalog number: CSB‐EL022397RA). In short, after preparing standard dilutions, samples, and reagents, we first added 100 μL of standard and sample per well and then the wells were incubated for 2 h at 37°C. Biotin‐anti GPx/CAT/SOD (100 μL; 1x) was added to each well and the wells were incubated for 60 min at 37°C. After washing the wells with 200 μL washing buffer, 100 μL of HRP–avidin (1x) was added to each well and incubated for 1 h at 37°C. TMB substrate (90 μL) was added to each well and incubated at 37°C for 30 min. Finally, after adding 50 μL of stop solution to each well and incubating (5 min at 37°C) the wells, the absorbance of the final mixture was read at 450 nm with an ELISA reader (Awareness Technology, Stat Fax ELISA reader, model number: 303 microwell readers; Wen et al., 2022).

### 2.10 Analysis of tissue‐related parameters

#### 2.10.1 Liver tissue lipid peroxidation levels

After isolating the liver tissues and washing with normal saline, the tissues were slowly homogenized with a homogenizer with the help of phosphate‐buffered saline (PBS). Then, to measure lipid peroxidation level of liver tissue, 100 μL of homogenized tissue was transferred to 50 μL of thiobarbituric acid reactive substances (TBARS) solution and after incubation at 37°C for 30 min and centrifugation (12,000*g* for 10 min), the absorbance of the final mixture was read at 532 nm with an ELISA reader (Awareness Technology, Stat Fax ELISA reader, model number: 303 microwell readers; Akbaribazm et al., 2020).

#### 2.10.2 Liver tissue total antioxidant capacity (TAC)

After preparing a homogenous sample of liver tissue, FRAP method was used to measure the TAC. Similar to what was stated in the FRAP assay section related to ALOF extract, here instead of the extract, 200 μL of homogenized liver was added to the FRAP solution and after incubation for 20 min at 25°C and centrifugation (12,000*g* for 10 min), the absorbance of the final mixture was read at 593 nm with an ELISA reader (Awareness Technology, Stat Fax ELISA reader, model number: 303 microwell readers; Akbaribazm et al., 2020).

#### 2.10.3 Liver tissue thiol levels

To measure the amount of total liver tissue thiol level, 250 μl of homogenous tissue was incubated with 12 μL of Tris‐EDTA (ethylenediaminetetraacetic acid) for 10 min at 25°C. The absorbance of tissue mixture was then read at 412 nm with an ELISA reader (Awareness Technology, Stat Fax ELISA reader, model number: 303 microwell readers; A1). Then, 20 μL of DTNB (Ellman's reagent; 5,5‐dithio‐*bis*‐[2‐nitrobenzoic acid]) was added to the liver tissue and after 15 min of incubation at 25°C, the absorbance of mixture was read with ELISA reader at 412 nm wavelength (A2). DNTB solution was selected as a blank and its absorbance was also read with ELISA reader at 412 nm wavelength (B). Then, the obtained numbers were put in the following formula (Samadi‐Noshahr et al., 2021).

$$\text{Total thiol concentration}\left(\mathrm{\mu M}\right)=\left(A2-A1-B\right)\times 1.07/\left(0.05\times 13.6\right).$$

### 2.11 Relative expression of PI3K, AKT, and mTOR by real‐time polymerase chain reaction (real‐time PCR) assay

#### 2.11.1 Total RNA extraction of liver tissues

Canvas biotech higher purity tissue total RNA purification kit (Canvas Biotech, Spain, catalog number: AN0152) was used to extract total RNA from liver tissue according to the manufacturer's protocol and guidelines. Briefly, 40 mg of liver tissue was transferred to a 1.5‐mL microcentrifuge tube. Then, 350 μL of buffer BLY (β‐mercaptoethanol added) was added to the tissue inside the tube. After incubation at 25°C for 5 min, the tissue mixture was transferred to the filter column in a 2‐mL collection tube and centrifuged (10,000*g* for 2 min). The resulting mixture was transferred to the clarified filtrate to a new 1.5‐mL microcentrifuge tube and 1 volume of 70% ethanol was added to it. The resulting mixture was then transferred to RNAprep spin column and centrifuged (10,000*g* for 90 s). After extracting the supernatant and washing the formed pellet twice with 250 μL of kit washing buffer, 50 μL of double deionized water was added to it and stored at −70°C. Nanodrop Thermo Scientific spectrophotometer (evaluation of the ratio of 260/280 nm; ND‐1000 UV–Vis Spectrophotometer) and 2% agarose gel electrophoresis were used to determine the purity and quality of the extracted RNA (Akbaribazm et al., 2020).

#### 2.11.2 cDNA synthesis and quantitative real‐time PCR (qPCR)

With the help of qScriber cDNA synthesis kit (qScriber highQu, catalog number: RTK0104), template cDNA was synthesized from the extracted RNA according to the protocol and guidelines of the manufacturer. cDNA synthesis mixture included 1000 ng of extracted RNA, 1 μL of oligo and random hexamer primers, 9 μL of DW, and 10 μL of qScriber reaction master mix. Gene sequences (Table 2) were designed with the help of version 6.5.52 gene runner software (Hastings Software), Primer‐3 software (http://primer3.wi.mit.edu), and then blasted with NCBI database (https://www.ncbi.nlm.nih.gov/tools/primer‐blast/). β‐Actin was selected as a housekeeping gene and the relative expression of genes was evaluated. The reproduction of the patterns was done with the help of Corbett Rotor thermocycler (Corbett Research, model number: Gene 6000 thermocycler) during 42 temperature cycles including presynthesis (2 min at 55°C), denaturation (5 min at 95°C), and annealing (1 min at 62°C), and extension (2 min at 72°C) stages. The qPCR mixture contained 1 μL of template cDNA, 10 μL of SYBR Green Master Mix, 1 μL of forward/reverse primers. After recording the threshold cycle (CT) of each gene and considering the CT control group and housekeeping gene in the fold formula change = 2^−∆∆CT^, the relative expression of genes was calculated (Akbaribazm et al., 2020).

$$\text{ΔΔCt}=\left(\left[\mathrm{Ct}\;\text{sample}-\mathrm{Ct}\;\text{housekeeping gene}\right]-\left[\mathrm{Ct}\;\text{sample}-\mathrm{Ct}\;\text{control}\right]\right).$$

**TABLE 2:** Primer sequences.

Gene	Sequences (5′–3′)
β‐Actin	Forward: AGGCATCCTCACCCTGAAGTA
	Reverse: CACACGCAGCTCATTGTAGA
Cas‐3	Forward: GTGGAACTGACGATGATATGGC
	Reverse: CGCAAAGTGACTGGATGAACC
Bcl‐2	Forward: TGTGGATGACTGACTACCTGAACC
	Reverse: CAGCCAGGAGAAATCAAACAGAGG
Bax	Forward: CGGCGAATTGGAGATGAACTGG
	Reverse: CTAGCAAAGTAGAAGAGGGCAACC
p53	Forward: CCTATCCGGTCAGTTGTTGGA
	Reverse: TTGCAGAGTGGAGGAAATGG
PI3K	Forward: AACACAGAAGACCAATACTC
	Reverse: TTCGCCATCTACCACTAC
mTOR	Forward: CCTGGCCACCGAGGATGAGC
	Reverse: CCAGCGTGGACCCTGCACTG
AKT	Forward: GTGGCAAGATGTGTATGAG
	Reverse: CTGGCTGAGTAGGAGAAC

### 2.12 Western blotting assay

To evaluate the effect of ALOF extract on the PI3K/AKT/mTOR pathway in liver tissue, the expression of the proteins of this pathway (PI3K, AKT, and mTOR) was evaluated by western blotting assay. Briefly, after isolating the liver tissues, washing with 200 μL of cold PBS and homogenizing with a homogenizer, 50 mg of the homogenized tissue was added to 500 μL of lysate buffer containing 0.1% NP‐40, 150 mmoL/L NaCl, and 50 mmoL/L Tris–HCl and was transferred and incubated at −4°C for 5 min. After centrifuging the resulting mixture (12,000*g* at −4°C for 10 min), 50 μg of it was added to the running buffer (containing 5 μL of 0.5% sodium deoxycholate, 5 μL of 62.5 mmoL/L Tris–HCl, 1 μL of β‐mercaptoethanol, 5 μL 10% glycerol) and transferred to SDS polyacrylamide gel (10%) and polyvinylidene fluoride (PVDF) membrane labeled with anti‐PI3K (catalog number; 9655, at 1:1000 dilution), anti‐AKT (catalog number; 9272, at 1:2000 dilution), and anti‐mTOR (catalog number; 2972, at 1:1000 dilution; Cell Signaling Technology) primary antibody and incubated (overnight at 4°C). Then, horseradish peroxidase‐conjugated secondary antibody (HRP‐linked antibody, Cell Signaling Technology, catalog number: 7074) was added to the primary antibody and after incubation (1 h at 37°C), the added antibody was blocked with the help of 200 μL bovine serum albumin, 500 μL of 0.05% Tween‐20, and Tris‐buffered saline. The bands were amplified with the help of enhanced chemiluminescence detection reagent (SignalFire ECL Reagent, Cell Signaling Technology, catalog number: 6883) and registered with version 1.3 Bio‐Rad software (Bio‐Rad Laboratories). Finally, the recorded bands were analyzed with the help of Image J software (Yuan et al., 2019).

### 2.13 Immunohistochemistry (IHC) assay

In evaluating the apoptosis/survival pathway of Bax/Bcl‐2/p53, the level of p53 protein expression in liver cells was evaluated. The rate (%) of p53‐ and Bcl‐2 positive cells/total cells was evaluated with a light microscope (Olympus CH3, Japan) equipped to camera system (Moticam Technologies) at 400X in 10 random fields of view. To identify and track p53‐ and Bcl‐2 positive cells, first, after separating the liver tissues and washing them with PBS, they were fixed in 10% formalin for 72 h. Then, the tissues were subjected to tissue routine process, paraffin blocks were prepared, and finally 5 μm sections were obtained from the blocks with the help of a Leica rotary microtome (Leica Biosystems, model number: RM2125 RTS). The slides were incubated for 24 h at 95°C. They were then incubated for 5 min in H_2_O_2_ (3%). After adding anti‐p53 monoclonal antibody (catalog number: sc‐98, Pab 1801, dilution 1:100) and anti‐Bcl‐2 (catalog number: sc‐509, dilution 1:200; Santa Cruz Biotechnology) and incubation (60 min at 25°C), HRP‐conjugated secondary antibody was added and finally the slides were incubated for 5 min at 25°C with 3,3′‐diaminobenzidine. PBS (1X) for 5 min was used to wash slides in each step and 5% bovine serum albumin was used as blocking buffer (Goodarzi et al., 2021).

### 2.14 Histopathological assay

Like what was stated in IHC assay, after preparing paraffin blocks from liver samples and 5 μm slices from them, hematoxylin and eosin (H&E) staining was done. Then, with a light microscope (Olympus CH3) equipped to camera system (Moticam Technologies) at 100X, histopathological evaluations were performed (Akbari et al., 2017).

### 2.15 Statistical analysis

In designing graphs, version 8 GraphPad Prism software were used. For statistical analysis, version 16 SPSS software (SPSS, IBM) was used to compare the mean differences of the data between groups by one‐way analysis of variance (ANOVA) test followed by Tukey's post hoc test after checking the normality between groups with the help of Kolmogorov–Smirnov test (*p* > .05). The *p* < .05 was considered to be significant between data and all data were reported as mean ± standard deviation (SD).

## 3 RESULTS

### 3.1 LD_50_ of ALOF extract

After 24‐h monitoring of the groups in terms of toxicity and lethality, it was determined that the lowest dose (*D*_L_) in which the rats suffered toxic symptoms or death was in the 5000 mg/kg group and the highest dose (*D*_S_) in which the rats did not show any toxic symptoms was in 1000 mg/kg group. Therefore, according to the formula of Lorke's method, the LD_50_ of ALOF extract is 2236 mg/kg (or 2.236 g/kg) and lower doses can be used in the study.

### 3.2 ALOF extract DPPH, FRAP, TFC, and TPC levels

Evaluation of polyphenolic, flavonoid, and antioxidant content of ALOF extract (with three repetitions) showed that the flower extract of this plant contained 189.6 ± 5.8 mg GAE/g polyphenol and 112.8 ± 4.4 mg RUE/g flavonoid and its DPPH radical scavenging activity 319.6 ± 6.6 μmol eq. Trolox/10 g and its total antioxidant capacity is 11426.4 ± 61.7 mmol Fe^2+^ mg (Table 3).

**TABLE 3:** ALOF extract TPC, DPPH, FRAP, and TFC levels.

ALOF extract parameters/unit	Value^a^
Total phenolic content (mg GAE/g dried plant)	189.6 ± 5.8
Total flavonoid content (mg RUE/g dried plant)	112.8 ± 4.4
DPPH (μmol eq. Trolox/10 g dried plant)	319.6 ± 6.6
FRAP (mmol Fe^2+^ mg dried plant)	11426.4 ± 61.7

^a^ Mean ± SD (*n* = 3).

### 3.3 Total body and liver weights of rats

**FIGURE 1:**
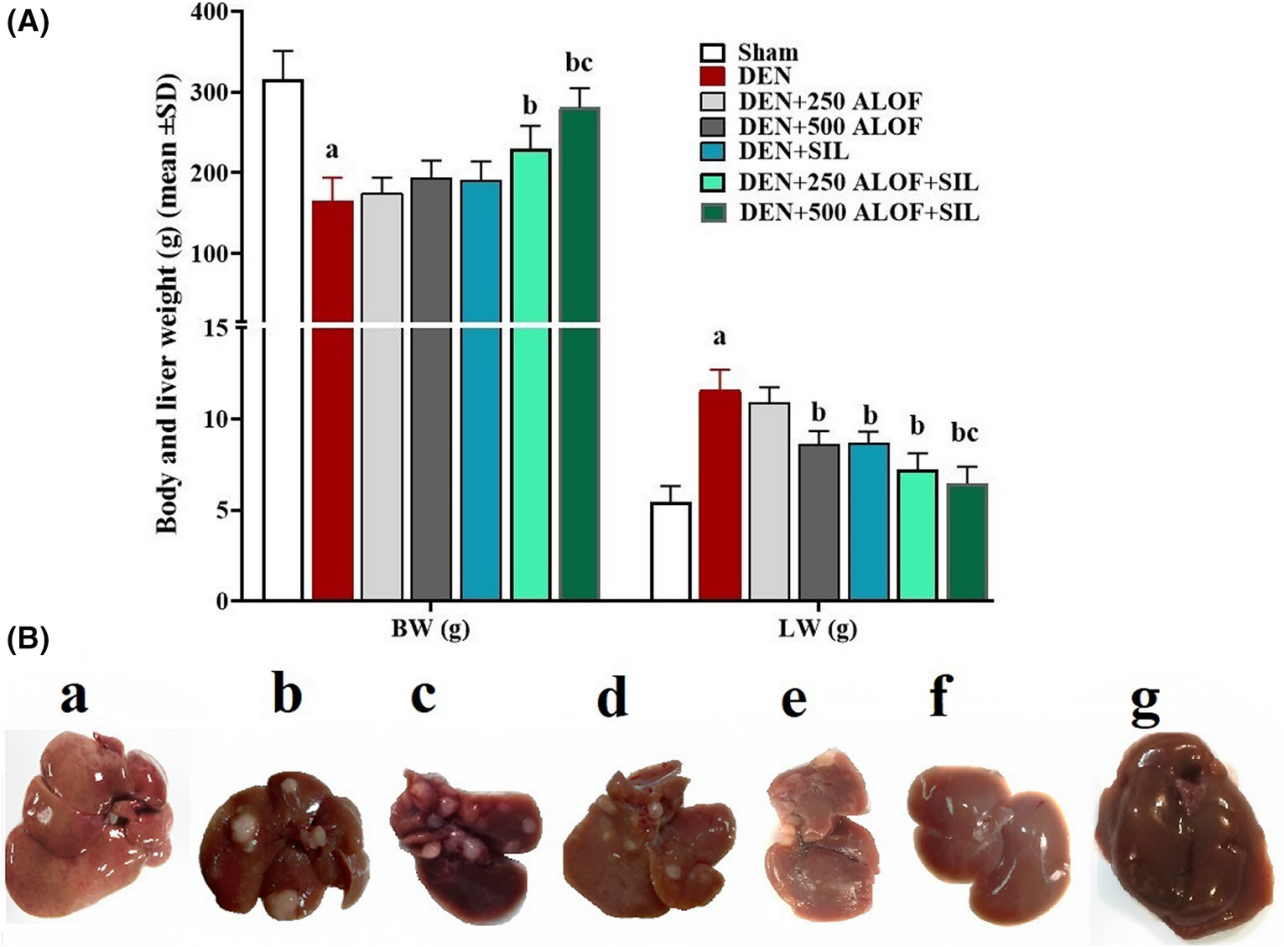
(A) Total body (BW) and liver (LW) weights (g; *n* = 10 rat/group, data are mean ± SD) and (B) macroscopic view of liver tissue in sham (500 μL PBS, a), diethylnitrosamine (DEN, b), treatment with silymarin (SIL; DEN + SIL, c), treatment with 250/500 mg/kg *Althaea officinalis* L. (ALOF) (DEN + 250 [d] and 500 [e] ALOF), and SIL cotreatment with 250/500 mg/kg ALOF (DEN + SIL + 250 [f] and 500 [g] ALOF) groups. ^a^(*p* < .01) comparison of DEN with sham, ^b^(*p* < .01) comparison of all treatment with DEN, and ^c^(*p* < .01) comparison of ALOF treatment with SIL.

Evaluating the total body weight (BW) and liver weight (LW), it was found that DEN significantly (*p* < .05) decreased BW and significantly (*p* < .05) increased LW compared to the sham group. In the SIL group (DEN + SIL), only the LW was significantly reduced compared to the DEN group, while in the SIL and 250 ALOF cotreatment group (DEN + 250 ALOF + SIL), both weight parameters were significantly (*p* < .05) improved compared to the DEN group. The most effective effect was observed in the cotreatment group of SIL and 500 ALOF (DEN + 500 ALOF + SIL), so that the BW in this group increased significantly compared to both DEN and DEN + SIL groups, and the LW in this group also significantly (*p* < .05) decreased compared to these two groups (Figure 1A). One of the reasons for the increase in liver weight in the groups receiving DEN is the high number of nodular inflammatory lesions on the liver, while the sham group did not have these nodular lesions and its weight was less than the other groups (Figure 1B).

### 3.4 Liver biochemical parameters

**FIGURE 2:**
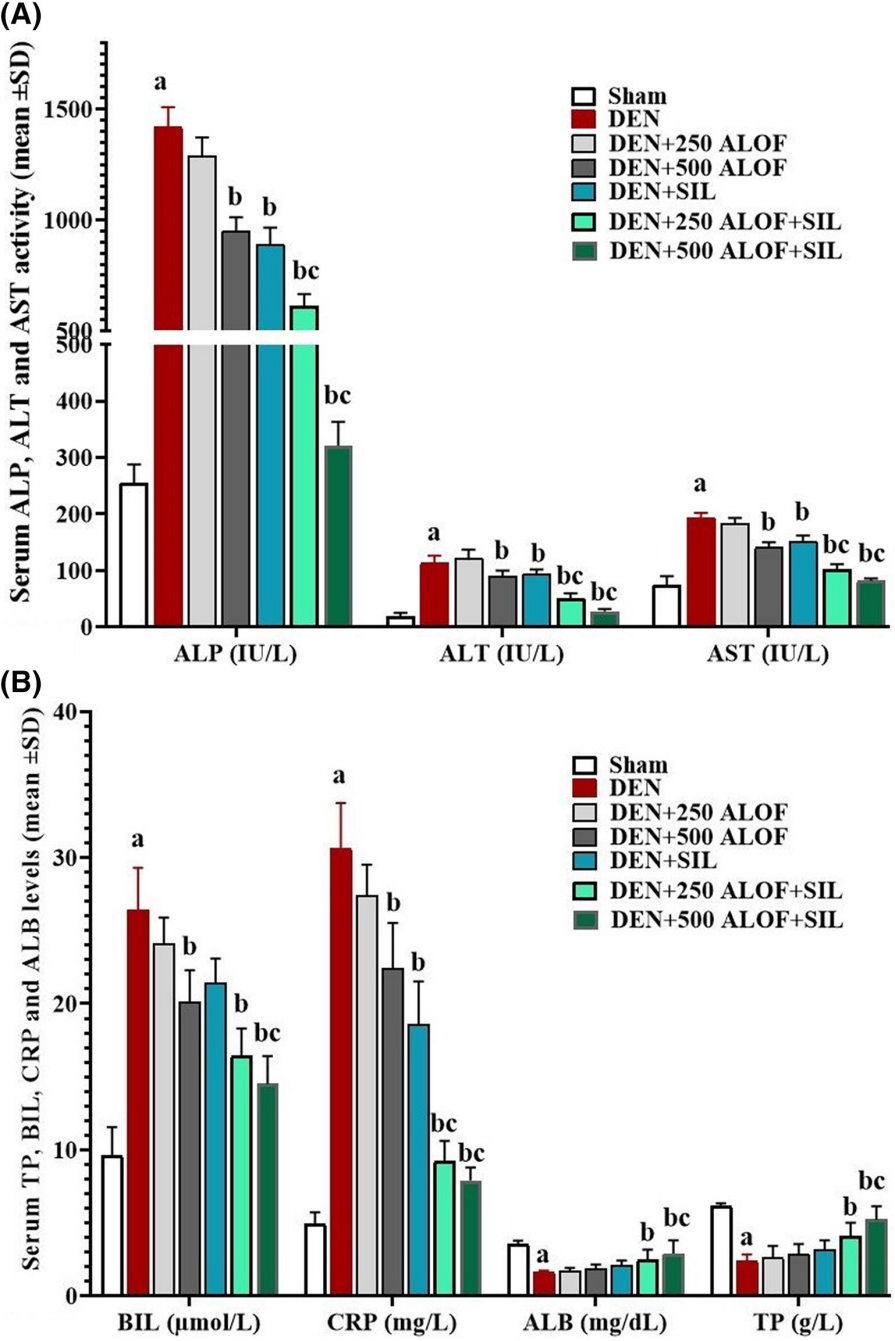
(A) Serum activity of alanine aminotransferase (ALT), aspartate aminotransferase (AST), and alkaline phosphatase (ALP) enzymes (IU/L) and (B) serum levels of albumin (ALB; mg/dL), total protein (TP; g/L), and bilirubin (BIL; μmol/L), C‐reactive protein (CRP; mg/L; *n* = 10 rat/group, data are mean ± SD) in sham (500 μL PBS), diethylnitrosamine (DEN), treatment with silymarin (SIL; DEN + SIL), treatment with 250/500 mg/kg *Althaea officinalis* L. (ALOF; DEN + 250 and 500 ALOF), and SIL cotreatment with 250/500 mg/kg ALOF (DEN + SIL + 250 and 500 ALOF) groups. ^a^(*p* < .01) comparison of DEN with sham, ^b^(*p* < .01) comparison of all treatment with DEN, and ^c^(*p* < .01) comparison of ALOF treatment with SIL.

After measuring the activities of serum liver enzymes (ALT, AST, and ALP), it was found that DEN significantly (*p* < .05) increased the activity of all three liver enzymes compared to the sham group. In the SIL and 500 ALOF treatment groups, the serum activity of all three liver enzymes significantly (*p* < .05) decreased compared to the DEN group. The maximum reduction in the activity of these enzymes was observed in the cotreatment groups (DEN + 250 and 500 ALOF + SIL), so that both cotreatment groups significantly (*p* < .05) decreased the activity of all three enzymes in comparison to the DEN and DEN + SIL groups (Figure 2A).

The results of serum BIL and CRP levels also showed that the levels of both factors were significantly (*p* < .05) higher in the DEN group than in the sham group. SIL was only able to significantly (*p* < .05) reduce the serum level of CRP compared to DEN group, while 500 ALOF alone was able to significantly (*p* < .05) reduce the level of both serum factors compared to DEN. In cotreatment groups, especially DEN + 500 ALOF + SIL, it was found that the serum level of BIL and CRP decreased significantly (*p* < .05) not only in comparison with DEN group but also in comparison with SIL (DEN + SIL) group (Figure 2B). The evaluation of serum ALB and TP levels between the groups also showed that DEN significantly (*p* < .05) reduced the levels of both factors. SIL and ALOF extract alone could increase serum ALB and TP levels, but this increase was not significant (*p* > .05). While in the DEN + 250 ALOF + SIL cotreatment group, the levels of both factors increased significantly (*p* < .05) compared to the DEN group. In the DEN + 250 ALOF + SIL cotreatment group, not only the levels of both factors significantly (*p* < .05) increased compared to the DEN group, but the levels of both factors also increased significantly (*p* < .05) compared to the DEN + SIL group (Figure 2B).

### 3.5 IL‐6, IL‐10, IL‐1β, and TNF‐α serum levels

**FIGURE 3:**
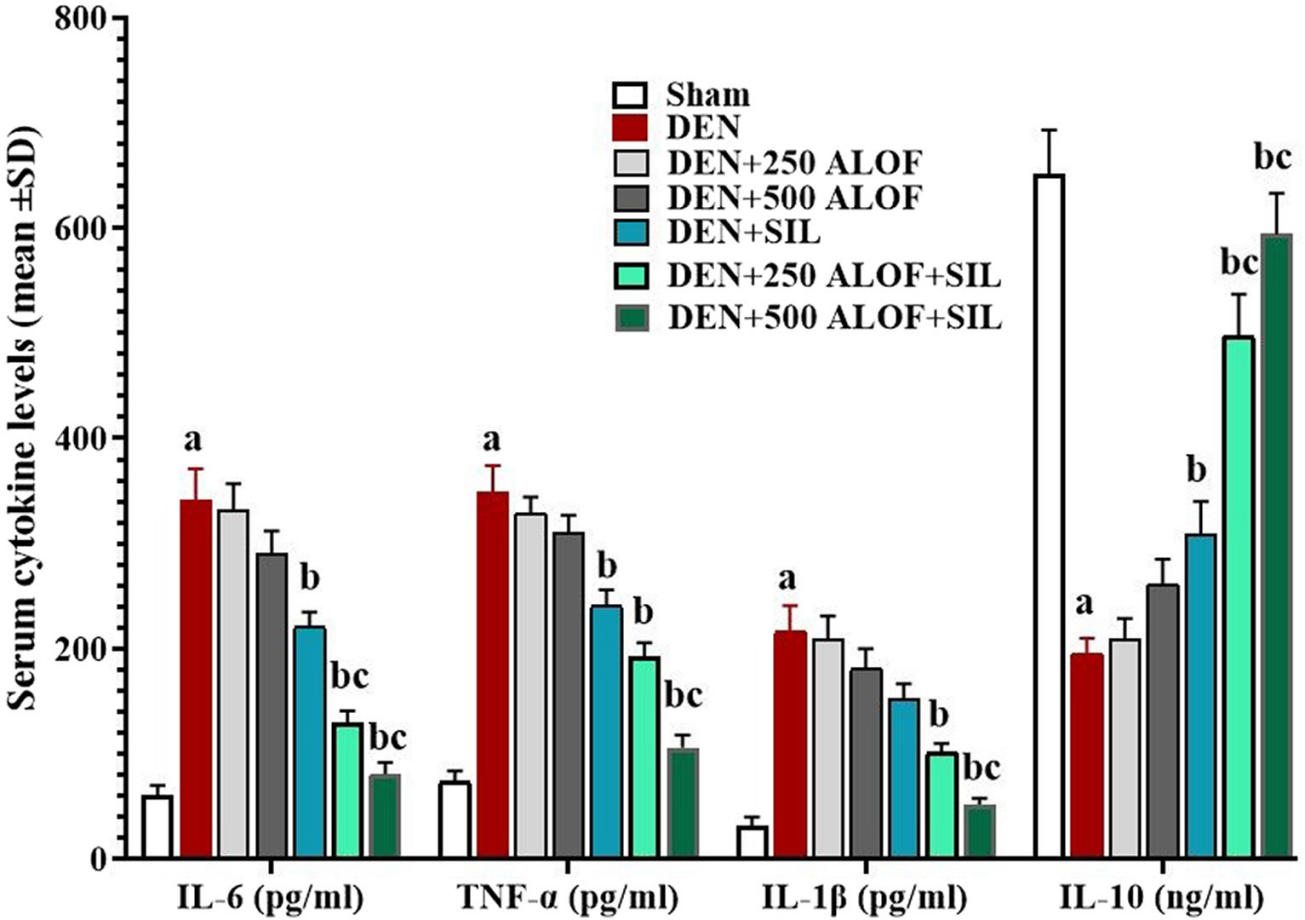
Serum levels of interleukin 10 (IL‐10; ng/mL), IL‐1β, IL‐6, and tumor necrosis factor alpha (TNF‐α; pg/mL; *n* = 10 rat/group, data are mean ± SD) in sham (500 μL PBS), diethylnitrosamine (DEN), treatment with silymarin (SIL; DEN + SIL), treatment with 250/500 mg/kg *Althaea officinalis* L. (ALOF; DEN + 250 and 500 ALOF), and SIL cotreatment with 250/500 mg/kg ALOF (DEN + SIL + 250 and 500 ALOF) groups. ^a^(*p* < .01) comparison of DEN with sham, ^b^(*p* < .01) comparison of all treatment with DEN, and ^c^(*p* < .01) comparison of ALOF treatment with SIL.

After measuring the levels of pro‐/anti‐inflammatory cytokines in the serum of the studied rats, it was found that DEN significantly (*p* < .05) increased the levels of all three proinflammatory cytokines (IL‐6, IL‐1β, and TNF‐α) compared to the sham group and also significantly suppressed (*p* < .05) the secretion of anti‐inflammatory cytokine IL‐10 compared to the sham group. SIL alone was able to significantly (*p* < .05) decrease the serum levels of IL‐6 and TNF‐α and also significantly (*p* < .05) increase the level of IL‐10 compared to the DEN group. Although in the group that received only ALOF extract, the level of cytokines improved, but these changes were not significant (*p* > .05). While in ALOF + SIL cotreatment groups, especially DEN + 500 ALOF + SIL, the serum levels of all three proinflammatory cytokines were significantly (*p* < .05) decreased compared to the DEN group and the SIL group, and the level of the anti‐inflammatory cytokine IL‐10 increased significantly (*p* < .05) compared to both groups (Figure 3).

### 3.6 Serum GPx, SOD, and CAT activity and also NO levels

**FIGURE 4:**
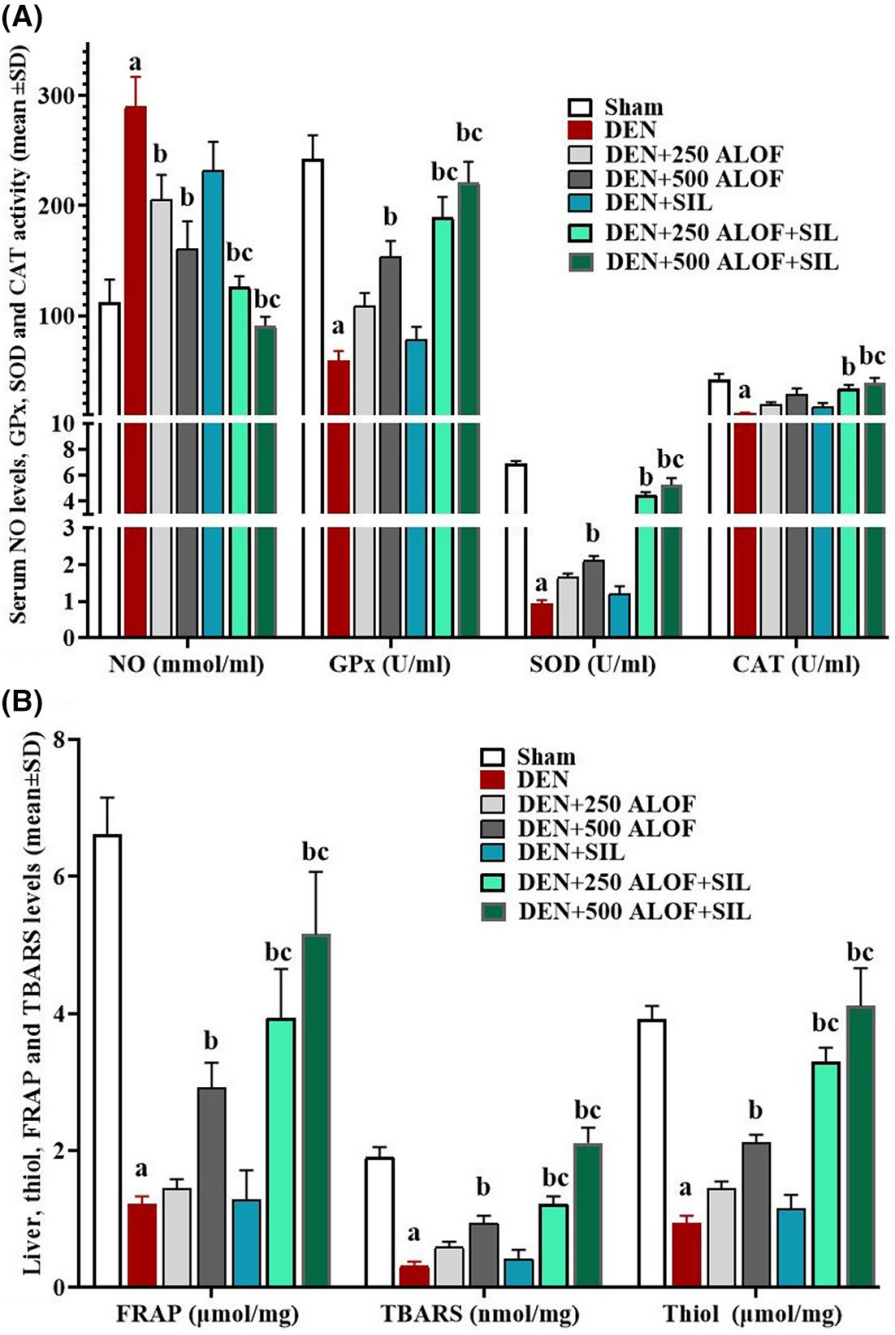
(A) Serum activity of glutathione peroxidase (GPx), superoxide dismutase (SOD), and catalase (CAT) enzymes (U/mL) alongside serum nitric oxide (NO) level (mmol/mL) and (B) the liver tissue levels of ferric reducing ability of plasma (FRAP; μmol/mg), thiol (μmol/mg), and thiobarbituric acid reactive substances (TBARS; nmol/mg; *n* = 10 rat/group, data are mean ± SD) in sham (500 μL PBS), diethylnitrosamine (DEN), treatment with silymarin (SIL; DEN + SIL), treatment with 250/500 mg/kg *Althaea officinalis* L. (ALOF; DEN + 250 and 500 ALOF), and SIL cotreatment with 250/500 mg/kg ALOF (DEN + SIL + 250 and 500 ALOF) groups. ^a^(*p* < .01) comparison of DEN with sham, ^b^(*p* < .01) comparison of all treatment with DEN, and ^c^(*p* < .01) comparison of ALOF treatment with SIL.

After measuring the activity of antioxidant enzymes (GPx, SOD, and CAT), it was found that DEN significantly (*p* < .05) reduced the activity of all three enzymes compared to the sham group. Although SIL alone was able to increase the activity of all three enzymes compared to the sham group, these changes were not significant (*p* > .05). While 500 ALOF alone could significantly (*p* < .05) increase the activity of GPx and SOD compared to the DEN group. The most changes in the activity level of all three endogenous antioxidant enzymes were observed in the cotreatment groups, especially DEN + 500 ALOF + SIL, so that in these groups, the activity level of all three enzymes compared to both DEN and SIL groups significantly (*p* < .05) increased (Figure 4A). In the evaluation of the results related to the serum NO level in different groups, it was also found that DEN caused a significant (*p* < .05) increase in its serum level compared to the sham group. ALOF in both 250 and 500 mg/kg groups (DEN + 250 and 500 ALOF) alone was able to significantly (*p* < .05) reduce the level of NO compared to the DEN group. Serum NO levels in both DEN + 250 and 500 ALOF + SIL cotreatment groups significantly (*p* < .05) decreased compared to both DEN and SIL groups (Figure 4A).

### 3.7 Liver tissue TAC, TBARS, and thiol levels

DEN caused a significant decrease in the antioxidant power and eventually significantly (*p* < .05) decreased the tissue level of FRAP, TBARS, and thiol compared to the sham group. Although SIL alone was able to increase the level of FRAP, TBARS, and thiol compared to the sham group, these changes were not significant (*p* > .05). While 500 ALOF alone could significantly (*p* < .05) increase the levels of all three tissue antioxidative parameters compared to the DEN group. The most changes in the level of all examined antioxidative parameters were observed in cotreatment groups (DEN + 250 and 500 ALOF + SIL), so that in these groups, the level of all three antioxidative parameters compared to both DEN and SIL groups significantly (*p* < .05) increased (Figure 4B).

### 3.8 Expression of liver PI3K, AKT, mTOR, p53, Cas‐3, Bcl‐2, and Bax genes

**FIGURE 5:**
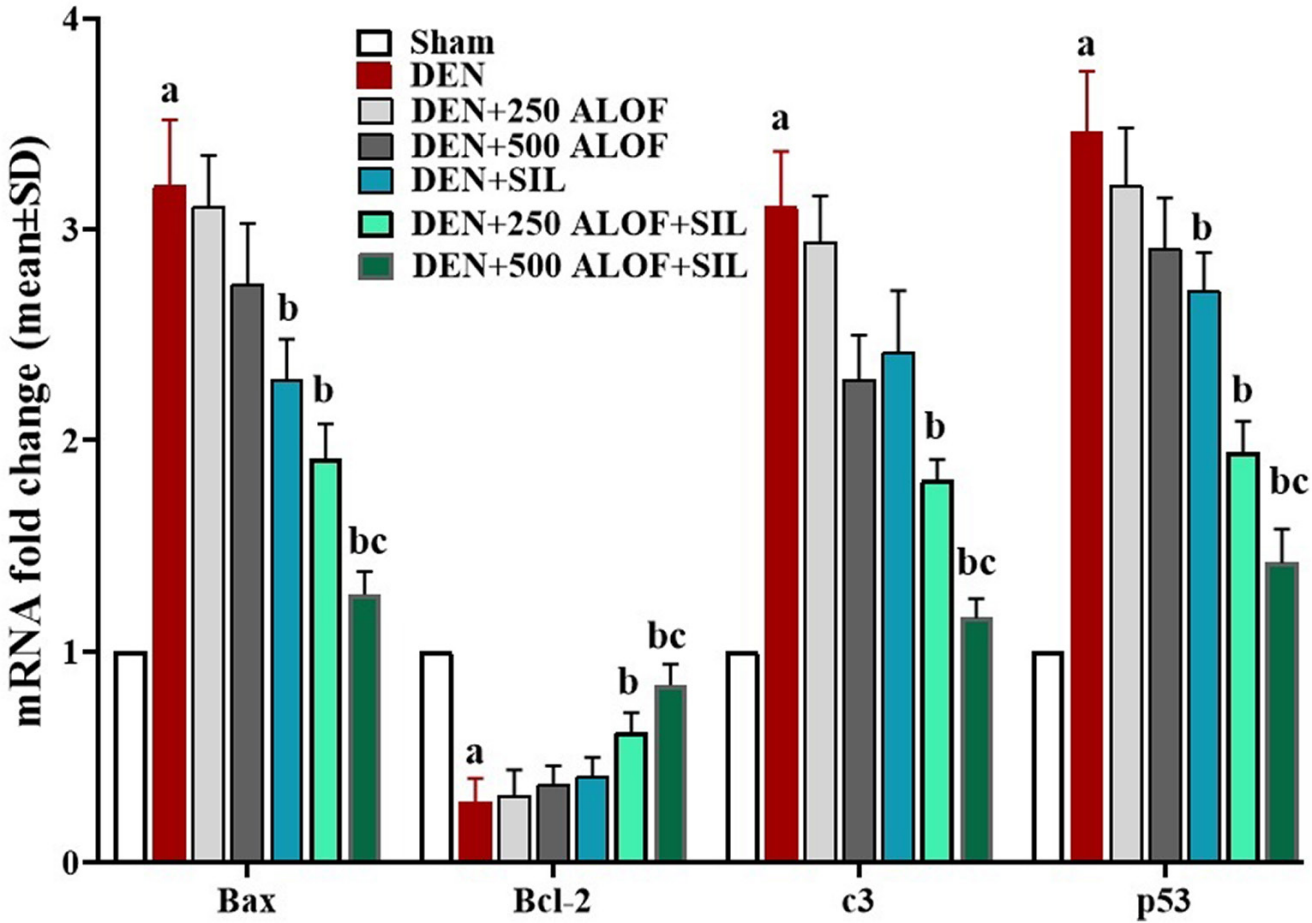
Cas‐3, p53, Bcl‐2, and Bax genes relative expression in liver tissue (*n* = 10 rat/group, data are mean ± SD) in sham (500 μL PBS), diethylnitrosamine (DEN), treatment with silymarin (SIL; DEN + SIL), treatment with 250/500 mg/kg *Althaea officinalis* L. (ALOF; DEN + 250 and 500 ALOF), and SIL cotreatment with 250/500 mg/kg ALOF (DEN + SIL + 250 and 500 ALOF) groups. ^a^(*p* < .01) comparison of DEN with sham, ^b^(*p* < .01) comparison of all treatment with DEN, and ^c^(*p* < .01) comparison of ALOF treatment with SIL.

Analysis of the results related to the expression of genes controlling apoptosis/survival pathways showed that DEN significantly (*p* < .05) increased the expression of proapoptotic genes (p53, Cas‐3, and Bax) and significantly (*p* < .05) suppressed the expression of antiapoptotic gene (Bcl‐2) in hepatocytes compared to the sham group. SIL alone could significantly reduce (*p* < .05) the expression of proapoptotic genes Bax and p53 compared to DEN. While in the DEN + 250 ALOF + SIL cotreatment group, the expression of all three proapoptotic genes significantly (*p* < .05) decreased and the antiapoptotic gene expression significantly (*p* < .05) increased compared to the DEN group. The most change was observed in the DEN + 500 ALOF + SIL cotreatment group so that in this group the expression of all three proapoptotic genes significantly (*p* < .05) decreased and the expression of antiapoptotic genes significantly (*p* < .05) increased compared to both DEN and SIL groups (Figure 5).

**FIGURE 6:**
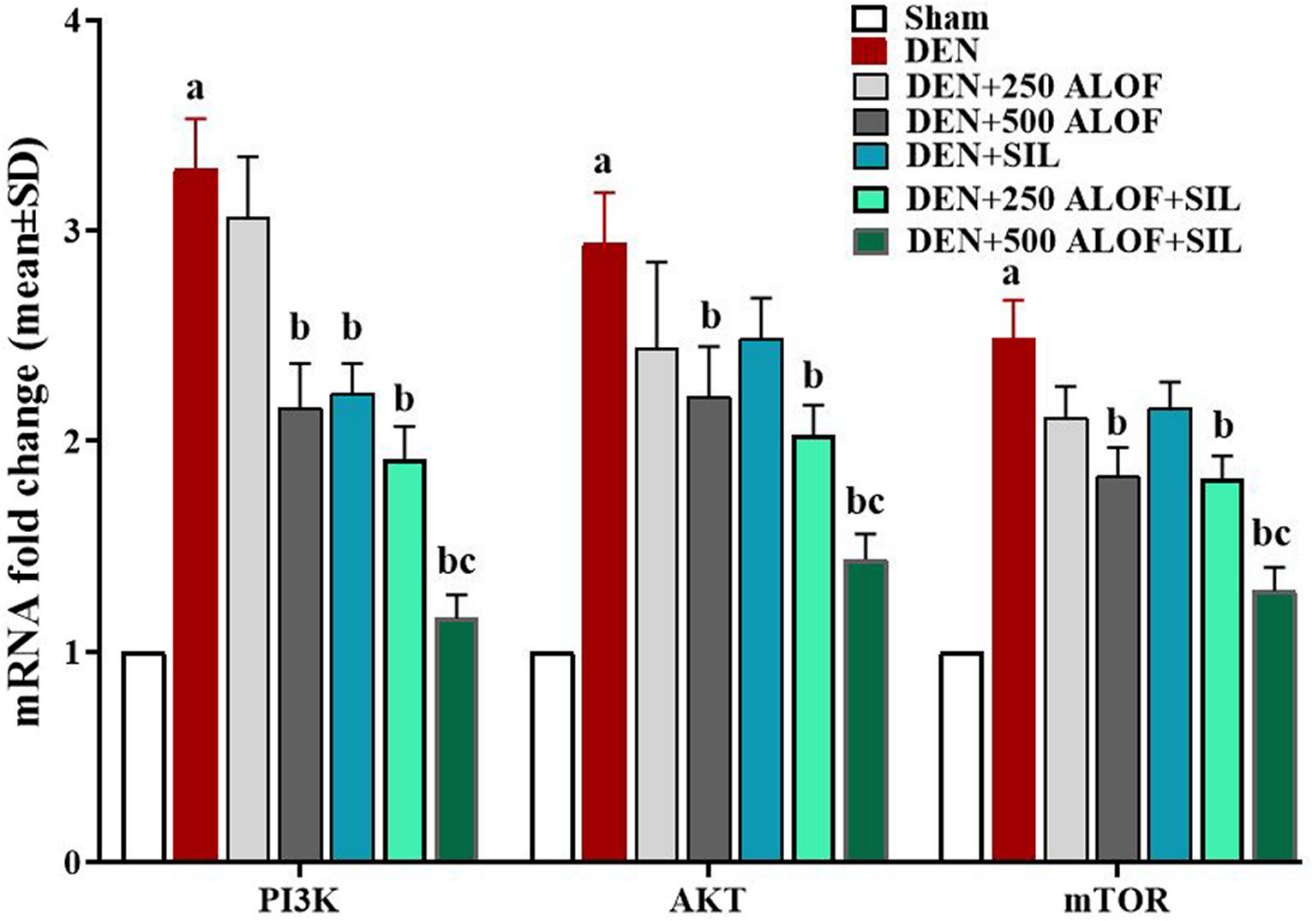
mTOR, AKT, and PI3K genes relative expression in liver tissue (*n* = 10 rat/group, data are mean ± SD) in sham (500 μL PBS), diethylnitrosamine (DEN), treatment with silymarin (SIL; DEN + SIL), treatment with 250/500 mg/kg *Althaea officinalis* L. (ALOF; DEN + 250 and 500 ALOF), and SIL cotreatment with 250/500 mg/kg ALOF (DEN + SIL + 250 and 500 ALOF) groups. ^a^(*p* < .01) comparison of DEN with sham, ^b^(*p* < .01) comparison of all treatment with DEN, and ^c^(*p* < .01) comparison of ALOF treatment with SIL.

After analyzing the results related to the expression of PI3K/AKT/mTOR signaling pathway genes, it was found that DEN stimulated this pathway and significantly (*p* < .05) increased all three genes of this pathway compared to the sham group. SIL was able to decrease the expression level of all three genes compared to the DEN group, but this decrease was significant (*p* < .05) only in the PI3K gene. The results showed that 500 mg/kg ALOF (in DEN + 500 ALOF group) alone could significantly (*p* < .05) reduce the expression level of all three genes of this pathway compared to DEN group. ALOF (250 mg/kg) was also able to significantly (*p* < .05) reduce the expression of all three genes of this pathway in the SIL cotreatment group compared to the DEN group (in DEN + 250 ALOF + SIL group). Meanwhile, 500 mg/kg ALOF also significantly (*p* < .05) reduced the expression of all three genes in the SIL cotreatment group (in DEN + 500 ALOF + SIL group) compared to both DEN and SIL groups (Figure 6).

### 3.9 Expression of liver PI3K, AKT, and mTOR proteins

**FIGURE 7:**
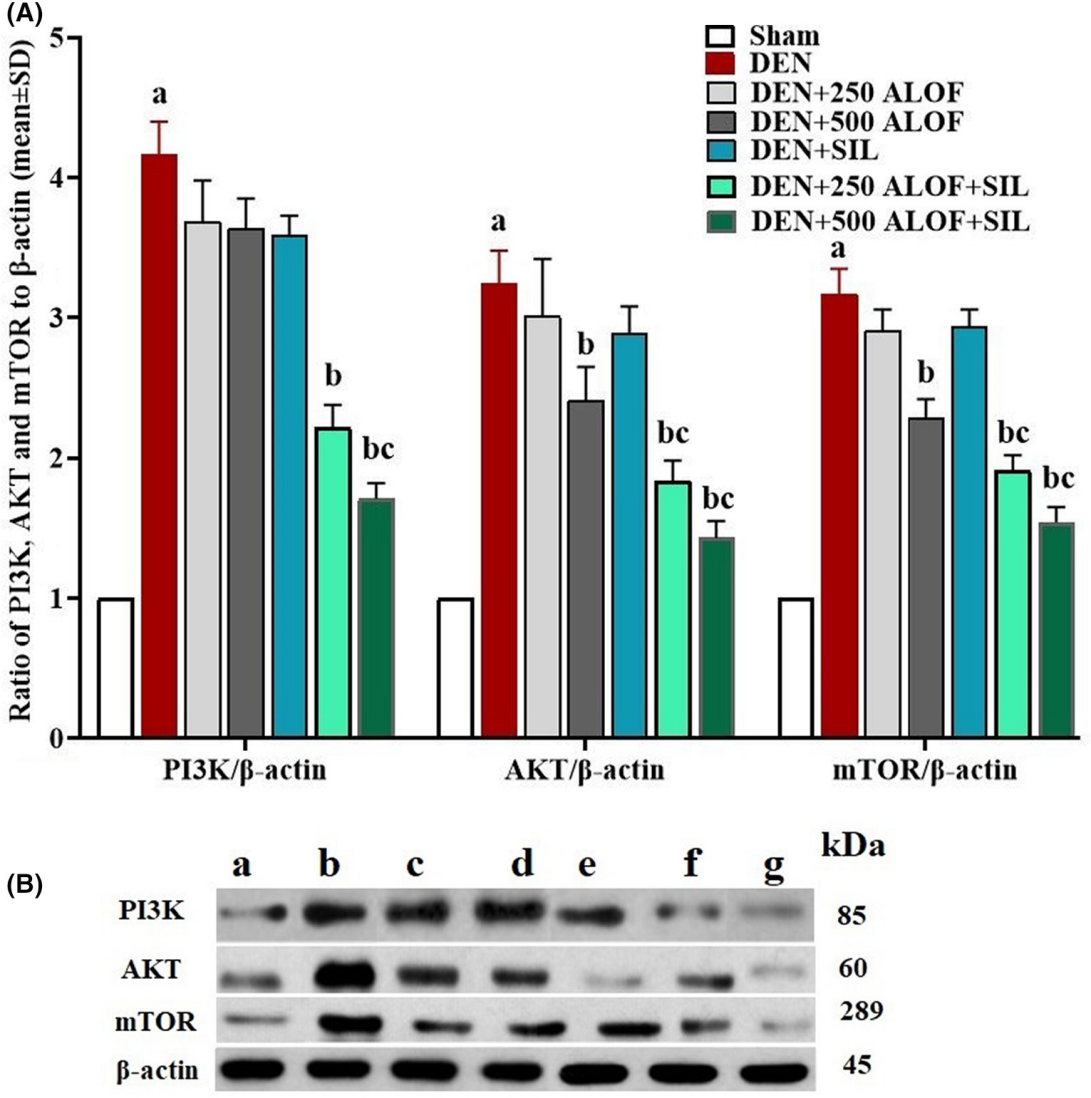
(A) mTOR, AKT, and PI3K proteins expression to β‐actin in liver tissue (*n* = 10 rat/group, data are mean ± SD) and (B) western blot bands in sham (500 μL PBS, a), diethylnitrosamine (DEN, b), treatment with silymarin (SIL; DEN + SIL, c), treatment with 250/500 mg/kg *Althaea officinalis* L. (ALOF) (DEN + 250 [d] and 500 [e] ALOF), and SIL cotreatment with 250/500 mg/kg ALOF (DEN + SIL + 250 [f] and 500 [g] ALOF) groups. ^a^(*p* < .01) comparison of DEN with sham, ^b^(*p* < .01) comparison of all treatment with DEN, and ^c^(*p* < .01) comparison of ALOF treatment with SIL.

The analysis of the results related to the expression of PI3K/AKT/mTOR signaling pathway proteins was also in line with the results related to the expression of the genes of this pathway. DEN strengthened this signaling pathway and significantly (*p* < .05) increased the expression of all three proteins of this pathway compared to the sham group. SIL alone decreased the expression of all three proteins of this pathway, but this decrease was not significant (*p* > .05). This was while 500 mg/kg ALOF (in DEN + 500 ALOF group) alone could significantly (*p* < .05) reduce the protein expression level of AKT and mTOR compared to DEN group. While ALOF, especially in 500 mg/kg along with SIL, was able to significantly (*p* < .05) reduce the expression of proteins of this pathway compared to both DEN and SIL groups (Figure 7).

### 3.10 Liver tissue p53‐ and Bcl‐2‐positive cells

**FIGURE 8:**
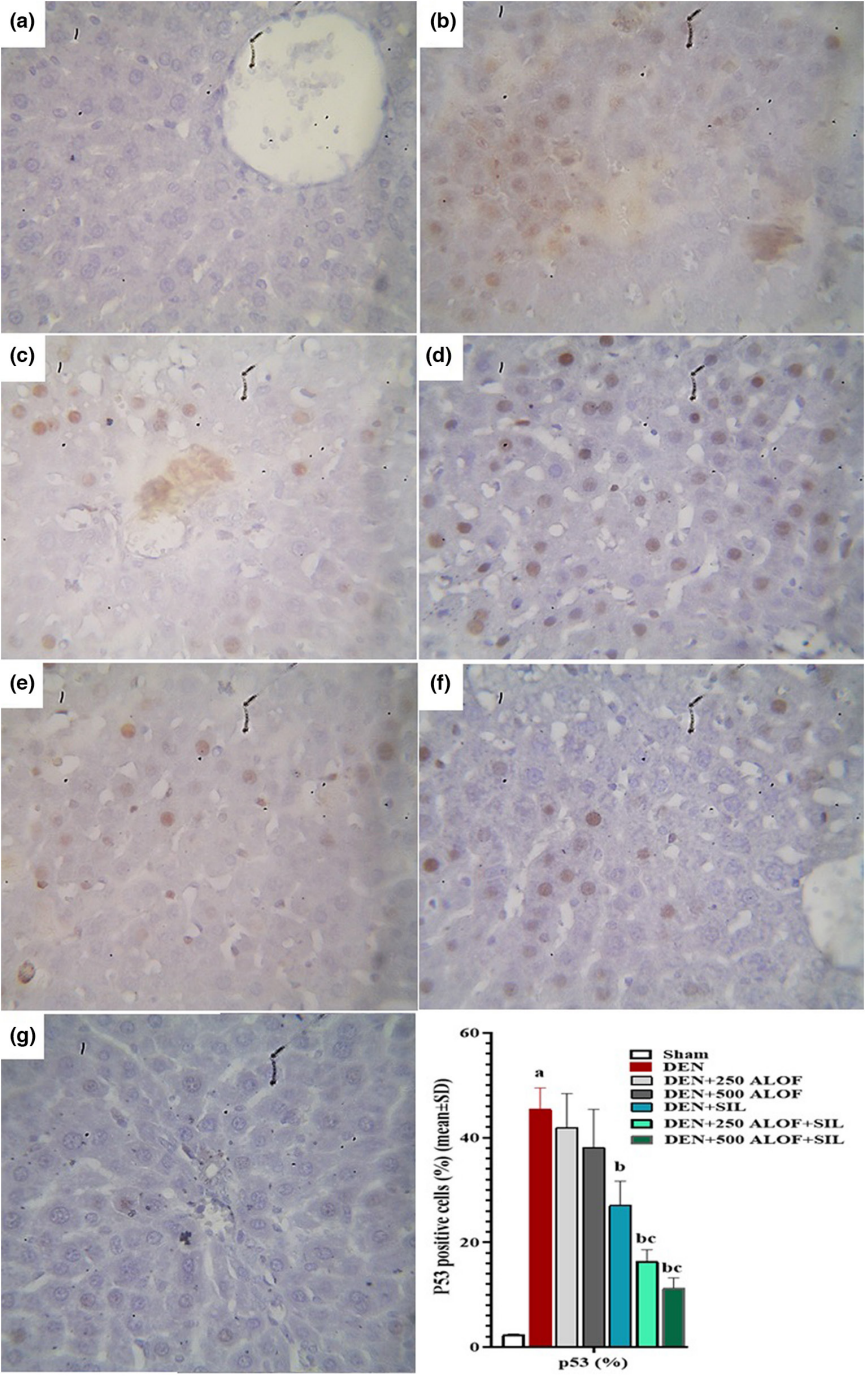
p53‐positive cells (%) in liver tissue by immunohistochemistry (*n* = 10 rat/group, data are mean ± SD) in sham (500 μL PBS, a), diethylnitrosamine (DEN, b), treatment with silymarin (SIL; DEN + SIL, c), treatment with 250/500 mg/kg *Althaea officinalis* L. (ALOF) (DEN + 250 [d] and 500 [e] ALOF), and SIL cotreatment with 250/500 mg/kg ALOF (DEN + SIL + 250 [f] and 500 [g] ALOF) groups. ^a^(*p* < .01) comparison of DEN with sham, ^b^(*p* < .01) comparison of all treatment with DEN, and ^c^(*p* < .01) comparison of ALOF treatment with SIL.

**FIGURE 9:**
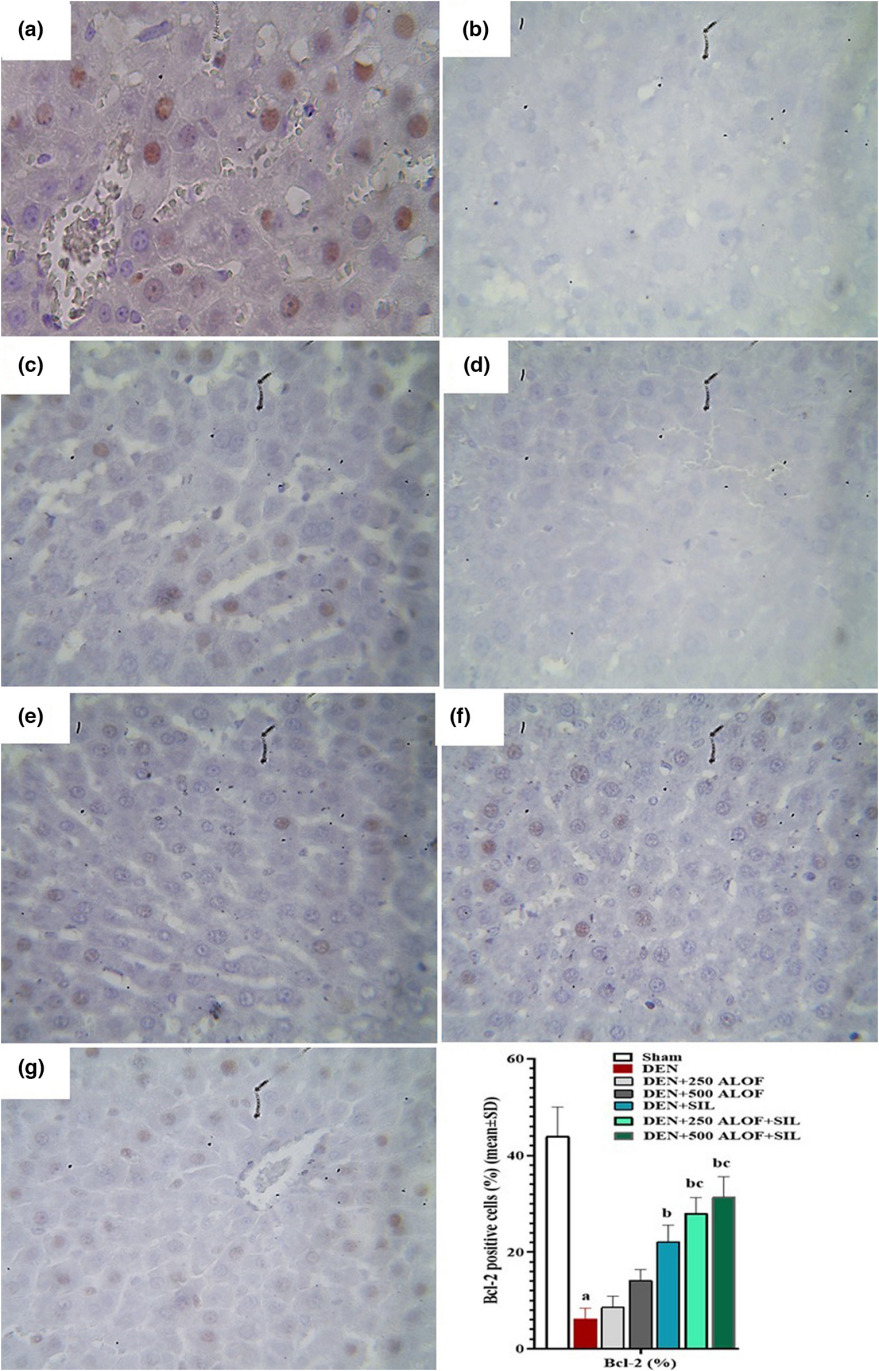
Bcl‐2‐positive cells (%) in liver tissue by immunohistochemistry (*n* = 10 rat/group, data are mean ± SD) in sham (500 μL PBS, a), diethylnitrosamine (DEN, b), treatment with silymarin (SIL; DEN + SIL, c), treatment with 250/500 mg/kg *Althaea officinalis* L. (ALOF) (DEN + 250 [d] and 500 [e] ALOF), and SIL cotreatment with 250/500 mg/kg ALOF (DEN + SIL + 250 [f] and 500 [g] ALOF) groups. ^a^(*p* < .01) comparison of DEN with sham, ^b^(*p* < .01) comparison of all treatment with DEN, and ^c^(*p* < .01) comparison of ALOF treatment with SIL.

The evaluation of the results related to the rate of p53‐ and Bcl‐2 positive cells showed that DEN caused a significant (*p* < .05) increase in the rate of p53‐positive cells and a significant (*p* < .05) decrease in the rate of Bcl‐2‐positive cells compared to the sham group. SIL alone caused a significant (*p* < .05) decrease in the rate of p53‐positive cells and a significant increase in the rate of Bcl‐2‐positive cells compared to the DEN group. The groups that received only 250 and 500 mg/kg ALOF extract also exhibited improved apoptotic indices (reduction of p53‐positive cells and increase of Bcl‐2‐positive cells) compared to the DEN group, but these changes were not significant (*p* > .05). While ALOF extract in both doses of 250 and 500 mg/kg in groups cotreated with SIL (in DEN + 250 ALOF + SIL group) caused a significant (*p* < .05) decrease in the rate of p53‐positive cells and a significant (*p* < .05) increase in the rate of Bcl‐2‐positive cells compared to both DEN and SIL groups (Figures 8 and 9).

### 3.11 Histopathologic findings

**FIGURE 10:**
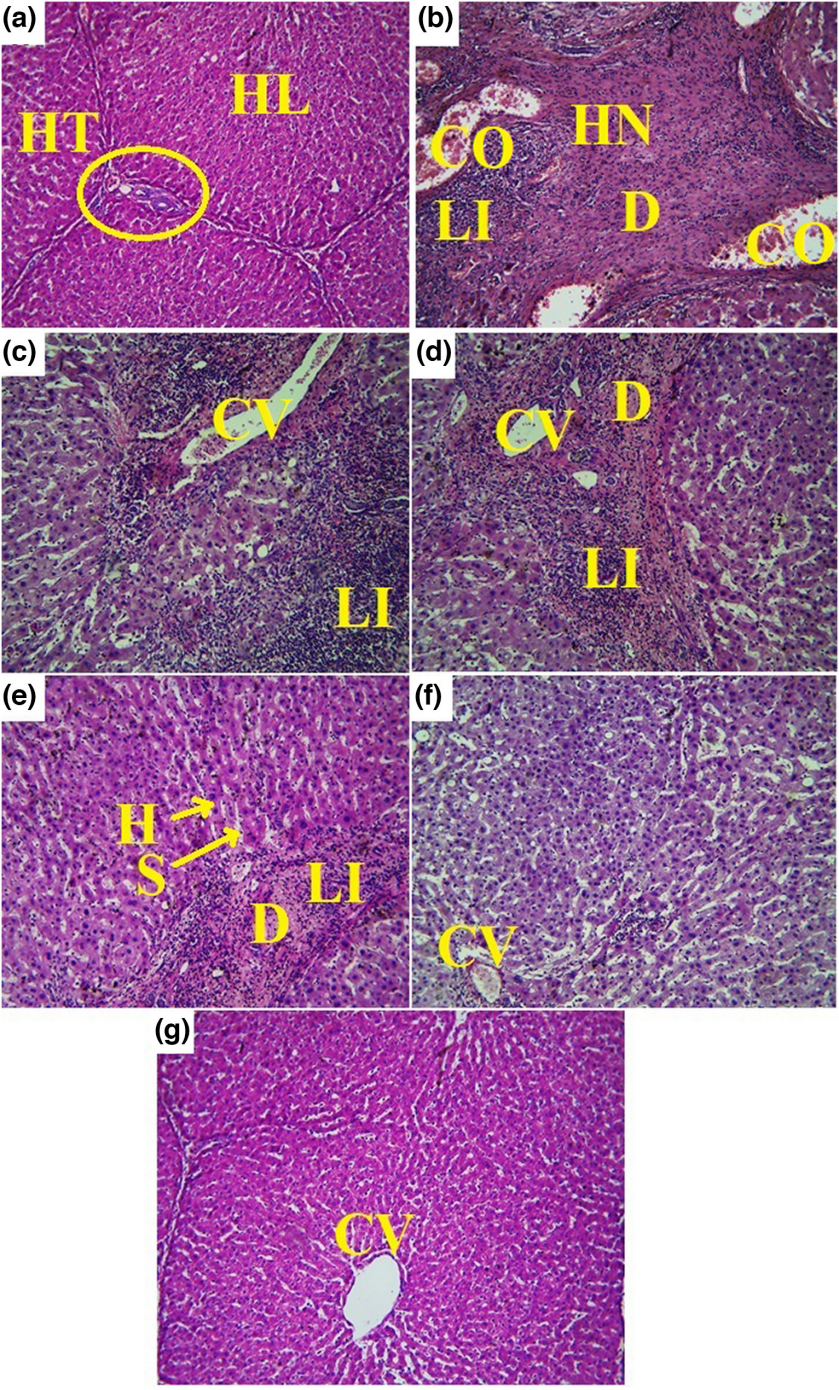
Histopathological changes in liver tissue in sham (500 μL PBS, a), diethylnitrosamine (DEN, b), treatment with silymarin (SIL; DEN + SIL, c), treatment with 250/500 mg/kg *Althaea officinalis* L. (ALOF) (DEN + 250 [d] and 500 [e] ALOF), and SIL cotreatment with 250/500 mg/kg ALOF (DEN + SIL + 250 [f] and 500 [g] ALOF) groups. Congestion (CO), hepatic nodules (HN), lymphatic infiltration (LI), central venule (CV), hepatic degeneration (D), hepatic sinusoid's (S), normal hepatic lobule (HL), hepatic triad (HT), and normal hepatocytes (H).

Histopathological evaluations of the liver tissue in the studied groups showed that DEN caused the formation of hepatic nodules (HN) consisting of degenerated hepatocytes (D) and lymphocytic infiltration (LI) next to the necrotic tissue. In this group, the normal hepatic lobule (HL) and hepatic triad (HT) were lost, and congestion (CO) and central venule (CV) dilatation were evident. In treatment groups with only SIL or ALOF extract, the volume of the formed HN was reduced to some extent fibrotic tissue and LI were visible next to CO. This was despite the fact that in cotreatment groups, especially 500 mg/kg ALOF, HL formation was normalized, and LI was greatly reduced. Also, the HN was not evident in the liver and the arrangement of hepatocytes (H) was clearly visible next to the liver sinusoids (S; Figure 10).

## 4 DISCUSSION

The results of this study showed that ALOF extract, along with SIL, can have synergistic effects through improving antioxidant parameters, suppressing inflammatory pathways, and regulating the apoptotic/survival pathways of PI3K/Akt/mTOR and Bax/Bcl‐2/Cas‐3/p53 for preserving the physiological function of hepatocytes and the structure of liver lobules against DEN‐induced hepatocellular carcinoma.

### 4.1 DEN‐induced HCC

DEN affects the physiological activity of hepatocytes through various mechanisms and induces the stages of tumorigenesis in them. The high production of ROS and RNS caused by its metabolism through cytochrome P450 of hepatocytes and its accumulation in the liver beyond the capacity of the endogenous antioxidant system causes damage to the polyunsaturated fatty acids of cell membranes, the structure of proteins, and the alkylation and methylation of DNA (Mo'men et al., 2020). Different studies have shown that metabolites of DEN inhibit the activity of antioxidant enzymes of cells (GPx, SOD, and CAT; Balamurugan & Karthikeyan, 2012; Shaban et al., 2013). Horng et al. (2017) studied the effects of DEN in inducing oxidative damage in rat liver and showed that this carcinogenic compound induces cell tumorigenesis through the DEN/ROS/PKCα/Rac‐1 axis and the inhibition of GSH, SOD, and CAT antioxidant enzymes. Also, in this study, it was found that the body weight of the rats decreased and the weight of the liver increased due to the presence of lesions caused by tumor formation (necrosis, fibrosis, and nodules; Horng et al., 2017). In another study, Pradeep et al. (2010) showed that DEN inhibited the activity of LPO, SOD, CAT, and GPx enzymes, and subsequently, serum NO and hepatic MDA levels increased as important indicators of the development of oxidative stress. Also, in this study, it was found that DEN increased the serum level of liver enzymes (ALT, AST, and ALP) and the level of BIL and CRP by disrupting the physiological function of hepatocytes (Pradeep et al., 2010). C‐reactive protein (CRP) is one of the most important inflammatory tests of the liver, especially in liver tumors, and its serum level increases when liver parenchyma (mainly hepatocytes) damage occurs (CRP synthesized primarily by the liver in response to inflammation), which is also used as an important indicator in animal studies. CRP in animal/human models (in vivo) can produce proinflammatory and inflammatory cytokines, especially TNF‐α and IL‐6, through stimulation of the p38 MAPK pathway. Although studies have shown that the serum level of rat‐source CRP is different from human‐source CRP, but the signaling pathways of both are common and the high level of both is considered as an indicator of the inflammatory phase (in humans, acute phase and in rats, chronic phase; De Beer et al., 1982; Hattori et al., 2003; Hotamisligil et al., 1996; Nabata et al., 2008; Xi et al., 2011; Zarubin & Han, 2005).

Studies show that DEN through JNK, P38, JAK, and STAT3 signaling pathways along with COX‐2, cyclin D1, and IGF‐2 mutation induces necrosis and apoptosis of hepatocytes and causes secretion of epidermal growth factor (EGF), platelet‐derived growth factor (PDGF), transforming growth factor beta (TGF‐β), and vascular endothelial growth factors (VEGFs), and subsequently tissue fibrosis and neoangiogenesis (Lin et al., 2016; Sivaramakrishnan & Devaraj, 2009). The results of this study also showed that DEN decreased antioxidant capacity (FRAP) and increased lipid peroxidation and liver proteins (TBARS and thiol) by inhibiting antioxidant enzymes (GPx, SOD, and CAT). Following the increase of ROS and RNS in this study, liver functional indices (ALB, TP, BIL, CRP, ALT, AST, and ALP) also changed.

DEN also stimulates the release of proinflammatory (IL‐6, IL‐1β, IL‐17, IL‐22, and TNF‐α) cytokines and inhibits anti‐inflammatory (IL‐10, IL‐13, and IL‐4) cytokines by strengthening various inflammatory pathways, including stimulating Kupffer cells, liver lymphocytes, and damaged cells. These cytokines induce and strengthen necrotic and apoptotic cascades in various tissues, including the liver. Studies have shown that DEN enhances the TNF‐α/NF‐κB, IL‐6/NF‐κB, and IL‐8/JAK 1, 2/STAT3 pathways. These signaling pathways play a key role in the survival/apoptosis of hepatocytes. Also, DEN induces the mitochondrial apoptosis pathway related to p53 through the increase of cycloxygenase‐2 (COX‐2, an inflammatory mediator effective in tumorigenesis; Chen et al., 2015; Kwon et al., 2010). PI3K/AKT/mTOR is one of the effective intracellular axes in regulating growth, metabolism, motility, and cell proliferation of tumor cells. As an inhibitor of various tumors, this axis is targeted by various drugs/compounds. This axis controls and leads inflammatory, apoptotic (effect on caspase 3 and p53), and oxidative cell cycle pathways, endo/exocytosis mechanisms, function of ATP pumps of the electron transport chain, synthesis of proteins, and glucose uptake of tumor cells (Miricescu et al., 2020; Yu et al., 2022). In addition, Sahin et al. (2014) showed that DEN strengthens the NF‐κB/cyclooxygenase‐2 pathway through the stimulation of the PI3K/Akt/mTOR and Nrf‐2/HO‐1 axis, and after tumorigenesis of hepatocytes and nodule formation, the function of these cells decreased and liver function indices changed (Sahin et al., 2014). In this study, in addition to strengthening the inflammatory pathways (increasing the level of IL‐6, IL‐1β, and TNF‐α), stimulating the mitochondrial apoptotic cascade (Bax/Bcl‐2/Cas‐3/p53) and PI3K/AKT/mTOR axis also occurred.

### 4.2 Anticancer effects of ALOF extract and synergistic effects with SIL

The results of this study showed that the LD_50_ of ALOF used in the study was 2236 mg/kg and its high polyphenolic and flavonoid contents recorded its high antioxidant capacity in DPPH and FRAP evaluations. The results showed that ALOF extract, both alone and together with SIL, synergistically and dose‐dependently improved the physiological function of hepatocytes' and maintained their normal structure through antitumor, anti‐inflammatory, and antioxidant pathways. ALOF extract was able to decrease the levels of liver enzymes (ALT, AST, and ALP), BIL, and CRP and increase ALB, TP both alone and together with SIL in a synergistic and dose‐dependent manner (especially in DEN + 500 ALOF + SIL group). Also, ALOF extract, especially in cotreatment groups with SIL in a dose‐dependent manner, inhibits inflammatory pathways (decrease in the level of IL‐6, IL‐1β, and TNF‐α and increase in IL‐10) and oxidative stress (increase in the activity of GPx, SOD, and CAT and decrease NO levels) induced by DEN.

Morovatisharifabad et al. (2020) in a study on the protective effects of ALOF in diazinon‐induced hepatotoxicity showed that the extract of this plant can lead to a decrease in the level of hepatic enzymes (ALT, AST, and ALP) and protect the structure and function of the liver against oxidative damage through the stimulation of endogenous antioxidant enzymes (GPx, SOD, and CAT; Morovatisharifabad et al., 2020). Sutovska et al. (2011) also showed that ALOF extract improved ovalbumin‐induced airways inflammation in guinea pigs by suppressing inflammatory pathways (decrease in the level of IL‐6 and IL‐1β; Sutovska et al., 2011). In the study of the protective effects of ALOF extract on indomethacin‐induced gastric ulcer in rats, Zaghlool et al. (2019) showed that this extract can reduce tissue MDA, thiol, and NO by increasing GPx and SOD activity.

Various studies show that this plant is rich of polyphenol compounds such as daidzein, genistein, apigenin, quercetin, kaempferol, catechin, resveratrol, curcumin, rutin, betulinic acid, and artemisinin. These compounds can be effective with their anti‐inflammatory, antiangiogenic, and antiproliferative effects on the growth, proliferation, metabolism, function, and motility of tumor cells (Dhanasekaran et al., 2009; Dhingra et al., 2022; Kaur et al., 2019). Quercetin is a flavonoid compound that affects the cell cycle, growth, metastasis, and proliferation of colon, breast, and prostate tumors through various pathways such as p53/miR‐34a/SIRT1, PI3K/AKT/mTOR, IL‐6/STAT3, and Notch/AKT/mTOR and suppresses tumor cells (Lou et al., 2015; Michaud‐Levesque et al., 2012). In addition, kaempferol present in this plant controls the cell cycle of tumor cells in G1 and G2/M phases by increasing the expression of p21, p27, p53, and Chk2 and causes them to arrest (Huang et al., 2013). Chodon et al. (2007) also showed that genistein inhibited the growth and proliferation of DEN‐induced HCC tumor cells through antioxidant pathways and control the mitochondrial apoptotic cascade and caspase‐3, which finally protected the physiological function and normal structure of liver tissue (Chodon et al., 2007).

## 5 CONCLUSION

Medicinal plants in pure form or effective substances extracted from them can be used as prodrugs/additives as low‐cost, simple, and ecofriendly agents in the pharmaceutical industry in the prevention/treatment of various tumors. According to the results of this study, it seems that ALOF extract can protects the physiological function of hepatocytes and preserve the normal architecture of liver lobules against oxidative/inflammatory damages caused by carcinogens. It is suggested that for complementary and clinical trial studies in humans, other possibly effective signaling pathways of ALOF extract were investigated in other models of hepatic damages. Furthermore, it is possible to use green synthesis or ALOF extract (or effective ingredients) loaded in nanocarriers in various animal tumor models (especially HCC) through signaling pathways related to the growth and metastasis of tumor cells.

## AUTHOR CONTRIBUTIONS

**Zhenqian Wang:** Conceptualization (equal); software (equal); supervision (equal). **Xiao Jiang:** Investigation (equal); methodology (equal); validation (equal). **Long Zhang:** Writing – original draft (equal). **Han Chen:** Conceptualization (equal); project administration (equal); supervision (equal).

## CONFLICT OF INTEREST STATEMENT

The authors declare that they do not have any conflict of interest.

## ETHICS STATEMENT

This article does not contain any studies with human participants performed by any of the authors. Maintaining, feeding, and sacrificing animals were done according to the national standards protocol and guidelines for the care of laboratory animals (NIH Publication 80‐23, 1996) approved by 905th Hospital of the Chinese People's Liberation Army Navy ethic committee.

## Data Availability

The data that support the findings of this study are available on request from the corresponding author. The data are not publicly available due to privacy or ethical restrictions.
